# Assessing the Origin of the COVID19 Pandemic Is Still Important

**DOI:** 10.1096/fba.2026-00227

**Published:** 2026-08-06

**Authors:** Jason D. Bannan

**Affiliations:** ^1^ Fairfax Virginia USA

**Keywords:** COVID19, laboratory origin, occupational infection, pandemic origin, SARS‐CoV‐2, zoonotic origin

## Abstract

The COVID19 pandemic, caused by the betacoronavirus SARS‐CoV‐2, now ranks among the world's deadliest plagues, and certainly one of the most significant public health crises in the last 100 years. Despite more than 6 years of investigating the pandemic, it has been difficult to assess the origin of the disease in the City of Wuhan, China. The Wuhan Municipal Public Security Bureau regretfully suppressed information exchanges between physicians early in the Wuhan outbreak, which interfered with the contact tracing necessary for epidemiological investigations. The scientific community and government agencies throughout the world have been divided in their assessments of the pandemic's origin in Wuhan China. In the 2021 intelligence assessment released by the U.S. Office of the Director of National Intelligence (ODNI), only the FBI assessed the possibility of a laboratory origin. By 2023 the U.S. Department of Energy (DOE) revised its assessment to acknowledge a lab origin was likely, and in 2025 the U.S. Central Intelligence Agency (CIA) acknowledged a similar view. There has been political polarization of views, condescension and accusations of conspiracies among scientists. The COVID19 pandemic is prompting policy changes to research guidelines and recommendations for research restrictions based upon the assessment that pandemic may have resulted from laboratory research. While some express this as politically motivated, as we approach the seventh year of the COVID19 pandemic, more evidence has emerged that supports the research origin theory and scant evidence supporting a zoonotic emergence. It is imperative that our policy makers understand the facts when considering legislation and policies that could affect infectious disease research.

## 1 Historical Precedence Supported a Zoonotic Origin of the COVID19 Pandemic

In early 2020 it was reasonable to assume that the emergence of the COVID19 pandemic in Wuhan, China was another zoonotic event [1, 2, 3, 4, 5, 6, 7]. There are seven coronaviruses known to infect humans: HCoV‐229E, HCoV‐HKU1, HCoV‐NL63, HCoV‐OC43, MERS‐CoV, SARS‐CoV, and SARS‐CoV‐2 [8]. Zoonoses are hypothesized for four of the human coronaviruses [9, 10, 11]. The most compelling evidence for zoonotic emergence of coronavirus diseases in humans comes from the 2002 emergence of Severe Acute Respiratory Syndrome (SARS) and the 2012 emergence of Middle East Respiratory Syndrome (MERS) [12, 13].

SARS‐CoV, the virus that caused the 2002 SARS outbreak, first emerged in humans in Guangdong province, China. People with severe respiratory infections were documented in hospitals in more than eleven different locations throughout the province over a period of several months [14]. Epidemiological investigations showed that several of these patients were exposed to animals infected with a similar virus while dining or working at restaurants and markets in close proximity to caged animals [15, 16]. Serological evidence suggested traders of infected animals had been exposed to SARS‐like coronaviruses [17]. There was sufficient epidemiological evidence that early human SARS cases had resulted from zoonotic exposure.

The emergence of the disease MERS also showed evidence of zoonotic exposure to animals carrying a betacoronavirus. Excluding hospital‐acquired or familial infections, most MERS cases occurred via exposure to dromedary camels infected with the coronavirus MERS‐CoV [18, 19]. Since the first documented cases of MERS, there have been more than 2600 cases reported in 27 countries and 950 associated deaths [20].

## 2 Wuhan Was Considered an Unlikely Source of SARS‐Like Viruses With Pandemic Potential

Following the 2002–2003 SARS pandemic, the Chinese Academy of Sciences' Wuhan Institute of Virology (WIV), in collaboration with EcoHealth Alliance (EHA), a U.S.‐based, non‐profit research organization, conducted serological studies to determine the zoonotic exposure risk of people living in areas where bats carry Sars‐CoV‐related viruses [21]. Only 2.7% of people living near the bat caves around Jining, in China's Southwestern province of Yunnan, exhibited serum responses suggestive of exposure to SARS‐like viruses. The researchers chose random blood donors from people in the city of Wuhan in Hubei province as their negative serum controls because Wuhan is “…more than 1000 kilometers from Jining and where inhabitants have a much lower likelihood of contact with bats due to its urban setting.” In their published strategy to assess the zoonotic spillover risk of bat SARS‐CoV‐related coronaviruses, the researchers believed Wuhan was far from the high‐risk areas studied [22]. Additional research by the WIV established that bats in Hubei Province are not expected to be a source of SARS‐CoV‐2‐related viruses [23].

## 3 No Potential Zoonotic Sources of SARS‐CoV‐2 Like Viruses Were Found in Wuhan or Other Regions of China

Sporadic cases of Severe Fever with Thrombocytopenia Syndrome (SFTS) prompted a 2017–2019 survey of live animals sold in Wuhan markets [24]. Live animals (farmed and wild caught) were identified at numerous shops across four markets in Wuhan in 2019. No SARS‐CoV‐related coronaviruses were detected in Wuhan market animals or traders. A 2022 study of wild animals in and around Wuhan also found no human coronaviruses and no SARS‐CoV‐2‐related viruses [25]. Viral genome surveys of farmed animals throughout China failed to identify any SARS‐CoV‐2‐related viruses [26, 27].

## 4 Public Health Surveillance Reported no SARS‐Like Illnesses Elsewhere in China Prior to the COVID19 Oubreak in Wuhan

By 2014 China's National Influenza Center (CNIC) and CCDC network included 408 laboratories and 554 sentinel hospitals nationwide [28]. China's Centers for Disease Control (CCDC) initiated a nationwide project for the surveillance and characterization of acute lower respiratory infections and shared information with the US CDC [29]. This network conducted nationwide surveillance of patients with acute respiratory infections between 2009 and 2013, including the identification of human coronaviruses (HCoV) [30]. No cases of SARS were reported other than the 2012 reports of six cases of SARS illnesses in miners working in Mojiang county, Yunnan, the etiology of which remains unknown [31]. Prior to the COVID‐19 outbreak in Wuhan, there were no reports of unusual respiratory illnesses or SARS cases in any other Chinese provinces including Yunnan, where the WIV identified bat viruses related to SARS‐CoV‐2.

The Wuhan‐market origin hypothesis suggests that intermediary host animals infected with bat SARS‐CoV‐2‐like viruses from Yunnan or Southeast Asia were transported over 1,000 miles for sale in Wuhan, without being sold in the numerous provincial markets in Yunnan, Guizhou, Guangxi, Sichuan, Hunan, and Chongqing that serve over 318 million people or 21% of China's population [32]. Public health surveillance networks reported no unusual respiratory disease in this densely populated region of China prior to the outbreak in Wuhan, and no animals in Wuhan were found to be a source of SARS‐CoV‐2.

## 5 Genome Stability of SARS‐CoV‐2 Suggested Virus Adaptation to Humans

Unlike the emergence of SARS and MERS, COVID19 appears to have emerged from one dominant genotype of SARS‐CoV‐2 in Wuhan in the autumn of 2019 [33]. Genetic adaptations are crucial to successful cross‐species transmission of a virus and the ability to cause disease in a new host [34, 35]. In 2002, SARS cases sporadically emerged over several months in multiple locations in Guangdong province. Significant changes occurred in the SARS‐CoV genome that facilitated adaptation to humans during the early phases of the SARS pandemic [36]. Similarly, multiple genotypes of MERS‐CoV have been isolated and likely result from exposure to different animal sources at different times and locations [37]. Despite more than 2600 reported cases, MERS has not become highly communicable.

In contrast to the early phases of SARS and MERS outbreaks, few adaptive mutations were observed in SARS‐CoV‐2 in the early months of the COVID‐19 pandemic [38]. Early mutations arose as the result of transmission between humans, but most did not impart an advantage to the virus. In contrast to SARS‐CoV and MERS‐CoV, SARS‐CoV‐2 appeared well adapted to infection and spread in humans as it emerged in Wuhan.

## 6 The WIV as a Potential Source of SARS‐CoV‐2 and Occupational Risk

For almost two decades, the WIV, in collaboration with the EHA and other researchers, had been conducting coronavirus research in China and elsewhere, amassing thousands of bat samples containing a rich gene pool of coronaviruses [39]. One of the stated aims of the research was to understand the genomic changes necessary for viral adaptation to different host species, including zoonotic transfer to humans. These investigators studied the genomic changes as viruses adapted to a new host by passage on human cells and in transgenic mice.

A 2017 doctoral dissertation, by a student at the University of the Chinese Academy of Sciences at the WIV, described the genetic system the student developed which allowed WIV researchers to manipulate coronavirus genomes “without leaving any trace” [40]. The dissertation and several WIV publications state that their research with bat SARS‐related coronaviruses, including viruses known to infect human cells, were conducted in Biological Safety Level 2 (BSL2) laboratories [4, 41] WIV/EHA grant progress reports to the US National Institutes of Health described experiments with genetically altered viruses showed increased infection of human cell cultures and increased pathogenicity in animals expressing the human Angiotensin Converting Enzyme (ACE2) cell receptor [42]. In WIV/EHA collaborator Ralph Baric's congressional testimony, he stated he cautioned the President and CEO of EHA, Peter Daszak, that a BSL2 laboratory is inadequate for working with SARS‐like viruses [43]. Zhengli Shi, the WIV's primary coronavirus researcher, informed Baric that their regulations allow research with replication competent bat viruses at BSL2, despite Baric's and other scientists' concerns that the experiments warranted higher levels of laboratory containment [44]. The U.S. State Department alleges that they were aware of problems in the BSL3 and BSL4 high containment suites at the WIV [45].

A 2019 thesis by another WIV student characterized bat coronaviruses from throughout China and described a new lineage of SARS‐CoV‐2 related viruses from bats in Yunnan Province [46]. In 2020, the WIV published the genomes of SARS‐CoV‐2 and RaTG13, a virus closely related to SARS‐CoV‐2. WIV authors published an addendum detailing more information about RaTG13 and the student's work on three other complete virus genomes and partial genome sequences sufficient to establish Yunnan bats were colonized by viruses later shown to be related to SARS‐CoV‐2 [47]. This thesis established that the WIV was in possession of bat samples known to contain SARS‐CoV‐2‐like viruses prior to the outbreak. However, a 2019 publication of the student's work neglected to include the genetic data of viruses related to SARS‐CoV‐2 [23]. Additional phylogenetic and geographic information confirms that SARS‐CoV‐2‐related viruses are endemic in bordering countries of Southeast Asia, a significant distance from Wuhan City [48].

In 2018, the EHA along with the WIV and others submitted a research proposal titled “Project DEFUSE: Defusing the threat of bat‐born coronaviruses,” to the U.S. Defense Advanced Research Programs Agency (DARPA) [49]. The proposal described plans to introduce furin cleavage sites into SARS‐like viruses. The SARS‐CoV‐2 genome is unique in that it is the only SARS‐related coronavirus with a furin cleavage site (FCS) in its Spike Protein gene [50]. The location of the SARS‐CoV‐2 FCS is precisely where the Wuhan‐U.S. researchers proposed to insert an FCS into SARS‐related viruses in their DEFUSE proposal. The 2018 DEFUSE proposal did not receive U.S. funding from DARPA. However, EHA's CEO Peter Daszak said in a 2024 congressional hearing that he had not asked the WIV if they had proceeded with the proposed research without their U.S. collaborators [51]. While there is no proof that SARS‐CoV‐2 was created by the WIV, they had demonstrated the capability to create chimeric viruses that appeared natural, and they proposed constructing a virus with features resembling SARS‐CoV‐2.

## 7 Conclusion

In the 6 years since the COVID19 pandemic began in Wuhan, there is very little direct evidence that supports the zoonotic origin theory. Most of the support for a zoonotic origin of the COVID19 pandemic is based upon the historical precedence of other human coronaviruses. Unfortunately, The Wuhan Municipal Public Security Bureau's early interference suppressed epidemiological contact tracing that could have identified potential infectious sources, and no zoonotic sources have been identified. More evidence has emerged that supports assessments that the COVID19 pandemic may have originated from research activities at the Wuhan Institute of Virology. The WIV's collection of bat samples represents a documented source of SARS‐CoV‐2‐related viruses in Wuhan, a significant distance from where they are endemic. WIV researchers conducted studies of the genetic changes necessary for adaptation of SARS‐related viruses to humans; manipulated genomes which resulted in increased infection of human cells and pathology in animals expressing the human receptor; proposed to construct viruses with genomic elements like those found in SARS‐CoV‐2; and conducted much of their research in laboratories that most experts agree lacked the sufficient level of biosafety for research of pathogens with pandemic potential. These are the open‐source facts that lead many to assess that it is likely the COVID19 Pandemic began in Wuhan resulting from research activities at the Wuhan Institute of Virology.

In 2017, the Department of Health and Human Services (HHS) under President Trump, published a “Framework for Guiding Funding Decisions about Proposed Research Involving Enhanced Potential Pandemic Pathogens” [52]. A 2024 policy titled “Oversight of Dual Use Research of Concern (DURC) and Pathogens with Enhanced Pandemic Potential (PEPP)” was drafted during the Biden administration and was to begin in 2025 [53]. However, on May 5, 2025, President Trump issued Executive Order 14292, directing the White House Office of Science and Technology Policy (OSTP) to coordinate drafting of new biosafety and biosecurity policies, rescinding previous administration policies [54]. As of this writing, OSTP has not completed this task. Political divisions and partisan politics have tainted pandemic origin assessments and detracted from an in‐depth review of US federal grant and contract practices and their adherence to biosafety and biosecurity policies and guidelines. In 2014, a federal funding moratorium paused GOF research involving influenza, SARS, and MERS viruses. A Federal Experts Security Advisory Panel (FESAP) was chartered and published a risk–benefit assessment with implementation guidance following several high‐profile biosafety incidents at U.S. federal research facilities [55, 56]. Perhaps it is time to re‐charter a FESAP to review U.S. federal grant and contract processes, including those funding international research collaborations, and make recommendations that will improve biosafety and biosecurity oversight of domestic and international research collaborations without hindering research progress.

## Author Contributions

Jason D. Bannan is the sole contributor to this manuscript.

## Disclosure

Disclaimer: The views represented in this manuscript are those of the author and do not represent the views of the Federal Bureau of Investigation.

## Conflicts of Interest

The author declares no conflicts of interest.

## Data Availability

No data sets were generated or analyzed for this manuscript.
